# Fort CnoX: Protecting Bacterial Proteins From Misfolding and Oxidative Damage

**DOI:** 10.3389/fmolb.2021.681932

**Published:** 2021-05-04

**Authors:** Emile Dupuy, Jean-François Collet

**Affiliations:** ^1^WELBIO, Brussels, Belgium; ^2^de Duve Institute, Université catholique de Louvain, Brussels, Belgium

**Keywords:** CNOX, GroEL and GroES, thioredoxin family proteins, oxidative stress, HOCl, holdase, chaperone

## Abstract

How proteins fold and are protected from stress-induced aggregation is a long-standing mystery and a crucial question in biology. Here, we present the current knowledge on the chaperedoxin CnoX, a novel type of protein folding factor that combines holdase chaperone activity with a redox protective function. Focusing on *Escherichia coli* CnoX, we explain the essential role played by this protein under HOCl (bleach) stress, discussing how it protects its substrates from both aggregation and irreversible oxidation, which could otherwise interfere with refolding. Finally, we highlight the unique ability of CnoX, apparently conserved during evolution, to cooperate with the GroEL/ES folding machinery.

## Introduction

The powerful oxidant hypochlorous acid (HOCl; the active ingredient of household bleach) is produced by neutrophils to kill invading bacteria (Hurst, 2012; Schürmann et al., 2017). HOCl exerts its bactericidal action, at least in part, by damaging cellular proteins, which results in massive unfolding (Hawkins and Davies, 1998; Hawkins et al., 2003; Winter et al., 2008). It is therefore not surprising that bacteria evolved dedicated chaperones to fight HOCl-induced protein aggregation.

In the last 20 years, several bacterial chaperones providing protection against HOCl have been identified (Goemans and Collet, 2019). In the model bacterium *Escherichia coli*, they include the proteins Hsp33, RidA, and CnoX (Jakob et al., 1999; Müller et al., 2014; Goemans et al., 2018b), as well as polyphosphate, an inorganic polymer synthesized from ATP (Gray et al., 2014). These chaperones function as holdases: they hold their substrates in a folding-competent conformation during stress (Hoffmann et al., 2004; Gray et al., 2014; Müller et al., 2014; Goemans et al., 2018b) and transfer them to ATP-dependent foldases for active refolding after stress (Hoffmann et al., 2004; Gray et al., 2014; Müller et al., 2014; not shown for RidA). Interestingly, Hsp33, RidA, and CnoX have in common to be converted into chaperones by HOCl. Hsp33 is activated via the oxidation of four zinc-binding cysteines residues (Jakob et al., 1999), which induces structural changes in Hsp33 and results in the exposure of hydrophobic surfaces for interaction with unfolded proteins (Graf et al., 2004; Groitl et al., 2016). RidA and CnoX are activated via a different mechanism; in both cases, it is the reversible chlorination of positively-charged residues that increases the hydrophobicity of their surface and turns these proteins into efficient chaperones (Müller et al., 2014; Goemans et al., 2018b).

In this short review, we summarize the current knowledge on CnoX, a protein that combines both a chaperone and a redox-protective function. We first present the key structural and biochemical features of this protein, taking *E. coli* CnoX (*Ec*CnoX; previously known as YbbN) as a model, before discussing how *Ec*CnoX participates in the proteostasis network under HOCl stress. Finally, we briefly review intriguing differences between CnoX homologs.

## CnoX Uniquely Combines a Thioredoxin Domain Fused to a TPR Domain

**FIGURE 1:**
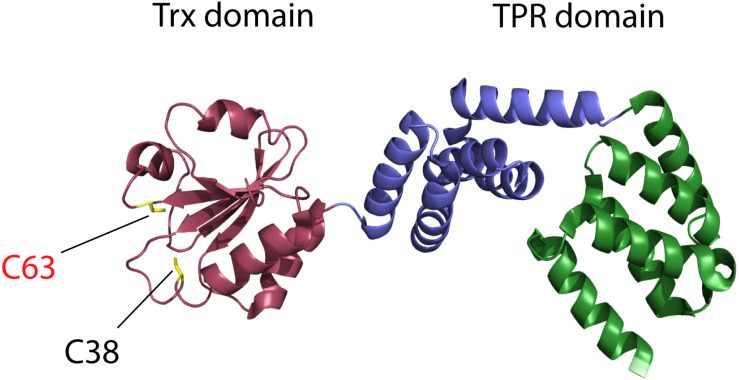
*Escherichia coli* CNOX (*Ec*CnoX) presents a thioredoxin domain fused to a tetratricopeptide (TPR) domain. The thioredoxin domain is represented in red, the first TPR subdomain in blue and the second TPR subdomain in green [PDB: 3QOU; (Lin and Wilson, 2011)]. Cys38 and Cys63 are shown in yellow: Cys38 is part of the typical catalytic motif of thioredoxins (SXXC motif in *Ec*CnoX) whereas Cys63 is involved in the formation of mixed-disulfide complexes with substrate proteins, thereby protecting them from irreversible oxidation.

It is the high homology of the N-terminal part of *Ec*CnoX to thioredoxin proteins that first drew the attention of researchers (Caldas et al., 2006). Proteins from the thioredoxin superfamily are found in most living organisms where they usually function as oxidoreductases. They share a conserved fold consisting of five β-strands surrounded by four α-helices (Pan and Bardwell, 2006; Collet and Messens, 2010) and display a conserved Cys–X–X–Cys catalytic motif. This motif undergoes oxidation-reduction cycles, allowing thioredoxins to catalyze disulfide-exchange reactions with substrate proteins. In *Ec*CnoX, the first cysteine of the canonical Cys–X–X–Cys motif is replaced by a serine (Ser_35_–X–X–Cys_38_). As a result, *Ec*CnoX does not function as an oxidoreductase; in contrast to active thioredoxins, it is unable to catalyze the *in vitro* reduction of insulin by dithiothreitol (Goemans et al., 2018b). When the structure of *Ec*CnoX (PDB: 3QOU) was solved (Lin and Wilson, 2011), it showed that a saddle-shaped tetratricopeptide (TPR) domain was fused to the C-terminus of the thioredoxin domain (Figure 1); TPR domains typically mediate protein-protein interactions (Allan and Ratajczak, 2011). In *Ec*CnoX, the TPR domain is composed of two similar subdomains with five α-helices each that define a groove rich in charged residues (Lin and Wilson, 2011).

## *Escherichia Coli* CnoX Is Turned Into a Chaperone by HOCl

Initial investigations suggested that *Ec*CnoX was a chaperone (Caldas et al., 2006), interacting with the essential foldase GroEL (Lin and Wilson, 2011), and with a potential role in heat shock response and/or DNA synthesis (Kthiri et al., 2008; Le et al., 2011). However, the exact function of this protein remained elusive. A few years ago, intrigued by the fact that the expression of *Ec*CnoX was induced by HOCl (Gray et al., 2013), we hypothesized that *Ec*CnoX was part of the defense mechanisms against this oxidant, which turned out to be true: we found that HOCl converts *Ec*CnoX into an efficient chaperone able to protect thermolabile proteins from aggregation, both *in vitro* and *in vivo* (Goemans et al., 2018b), and that this activity is required for *E. coli* survival under HOCl stress (Goemans et al., 2018b). Chaperone activation results from the reversible N-chlorination of several basic residues in the TPR domain, which increases the affinity of this region for unfolded polypeptides (Goemans et al., 2018b).

## *Escherichia Coli* CnoX Is More Than a Chaperone: It Is a Chaperedoxin

*Escherichia coli* CnoX is however more than a chaperone: it also protects cysteine residues in substrate proteins from irreversible oxidation. Upon oxidative stress, cysteine residues are indeed oxidized to sulfenic acids (–SOH), which are highly unstable and can be further oxidized to sulfinic (–SO_2_H) and sulfonic acid (–SO_3_H), two irreversible modifications (Gupta and Carroll, 2014). Interestingly, we found that a surface-exposed cysteine residue (Cys_63_) located in the thioredoxin domain of *Ec*CnoX, away from the SXXC motif, is involved in the formation of mixed-disulfide complexes with substrate proteins under HOCl stress (more than 130 proteins were identified), thereby protecting them from irreversible damage which could otherwise block reactivation (Goemans et al., 2018b). Thus, *Ec*CnoX uniquely provides dual protection against HOCl to its substrates: it prevents protein aggregation through the holdase function of its TPR domain while protecting sensitive cysteines from irreversible oxidation through its thioredoxin domain. Because it combines a chaperone function and a redox protective function, *Ec*CnoX was called a chaperedoxin (Goemans et al., 2018b). The reduction of the mixed-disulfides between *Ec*CnoX and its substrates after stress depends on glutathione (Goemans et al., 2018b), an abundant tripeptide that functions as a redox buffer and is mostly present in its reduced form (GSH) under normal conditions (Chesney et al., 1996).

## *Escherichia Coli* CnoX Functions With the GroEL/ES System

As a holdase, CnoX protects its substrates from aggregation under stress; it is however unable to help them regain their native conformation after stress. To that purpose, like most holdases, CnoX transfers its substrates to ATP-dependent foldases (Goemans et al., 2018b). In *E. coli*, two major folding machineries, the DnaK/J/GrpE and GroEL/ES systems, maintain protein homeostasis in the cytoplasm (Kerner et al., 2005; Calloni et al., 2012). It is interesting to note that these systems are inactive during HOCl stress because of the oxidation of essential residues and the drop in intracellular ATP levels (Barrette et al., 1987; Khor et al., 2004; Winter et al., 2005). We found that, like Hsp33 and polyphosphate, *Ec*CnoX cooperates with DnaK/J/GrpE (Hoffmann et al., 2004; Gray et al., 2014; Goemans et al., 2018b). However, in contrast to the chaperones above, *Ec*CnoX is also able to transfer its substrates to the essential GroEL/ES chaperonin (Goemans et al., 2018b), which makes *Ec*CnoX unique among holdases and raises a number of intriguing questions that we discuss below. Further highlighting the functional relationship between *Ec*CnoX and GroEL/ES, GroEL/ES obligate substrates are over-represented in the proteins found in a mixed-disulfide complex with *Ec*CnoX (Goemans et al., 2018b).

## Our Working Model

**FIGURE 2:**
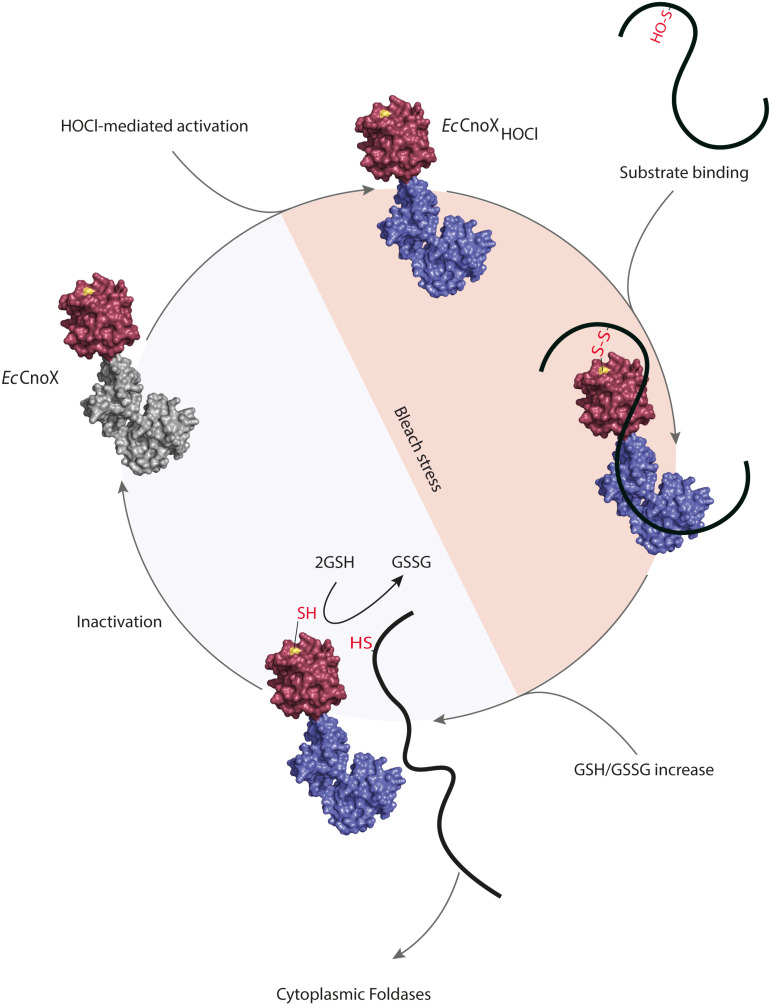
*Escherichia coli* CNOX, a hypochlorous acid (HOCl)-activated holdase with a redox protective function. Chlorination of residues in the C-terminal TPR domain of *Ec*CnoX by HOCl turns *Ec*CnoX into an efficient holdase. Cys68, located in the N-terminal thioredoxin domain, forms mixed-disulfide bonds with sensitive cysteines in substrate proteins, thereby protecting them from over-oxidation. After stress, normal GSH/GSSG ratios are restored, allowing the release of substrates from the mixed-disulfides and their transfer to foldases for ATP-dependent refolding. *Ec*CnoX is then inactivated, likely by thiol-based reducing pathways such as the thioredoxin and/or glutaredoxin systems. The surface of the thioredoxin domain is shown in red with Cys63 in yellow. The TPR domain is in gray when inactivated and in blue when activated upon chlorination.

By joining the pieces of the *Ec*CnoX puzzle, we came to the following model (Figure 2). Under HOCl stress, the intracellular ATP levels drop (Barrette et al., 1987) and glutathione is oxidized (GSSG) (Chesney et al., 1996). In parallel, chlorination of residues in the C-terminal TPR domain of *Ec*CnoX increases surface hydrophobicity, allowing *Ec*CnoX to interact with unfolded polypeptides in order to keep them in a folding competent conformation. At the same time, a cysteine (Cys_63_) located in the N-terminal thioredoxin domain of *Ec*CnoX forms mixed-disulfide bonds with oxidation-prone cysteines in substrate proteins, thereby protecting them from over-oxidation. Thus, *Ec*CnoX provides a solution to two threats proteins face. After stress, normal GSH/GSSG ratios are restored at the expense of NADPH (Chesney et al., 1996) and ATP levels are replenished (Gray et al., 2014), triggering the release of substrates from the mixed-disulfides and their transfer to foldases for ATP-dependent refolding. The inactivation of *Ec*CnoX most likely involves the cytoplasmic reducing pathways.

## CnoX Proteins Are Conserved in Most Gram-Negative Bacteria

CnoX is widely conserved in bacteria, being found in representatives of the proteobacteria, bacteroidetes, cyanobacteria, and many other phyla (Goemans et al., 2018a). Intriguingly, CnoX homologues are also present in species that are unlikely to encounter HOCl in their natural habitats, such as the non-pathogenic aquatic α-proteobacterium *Caulobacter crescentus* (*Cc*CnoX). Investigating the properties and function of *Cc*CnoX, we found that *Cc*CnoX combines, like *Ec*CnoX, holdase, and redox functions. Further, *Cc*CnoX conserves the ability to transfer its substrates to GroEL/ES for refolding. However, despite these crucial similarities, the two proteins show marked differences. First, because the surface of the TPR domain of *Cc*CnoX is more hydrophobic, the chaperone function is constitutive and does not need to be activated by HOCl, which allows *Cc*CnoX to protect substrate proteins from aggregation during thermal stress (Goemans et al., 2018a). Second, *Cc*CnoX harbors a classical CXXC catalytic motif in its N-terminal thioredoxin domain (Goemans et al., 2018a). As a result, *Cc*CnoX functions as an oxidoreductase and contributes to maintaining intracellular redox homeostasis in *C. crescentus* instead of protecting substrates from overoxidation under specific stress conditions. Thus, these data suggest that the structural and redox properties of CnoX proteins have been tailored during evolution to meet the needs of their host species.

## Conclusion and Remaining Questions

Two major conclusions can be drawn from the work summarized above. First, despite differences in how they exert their functions, *Ec*CnoX and *Cc*CnoX have in common to combine a chaperone and a redox function, which suggests that this property is conserved among the family of CnoX proteins. While further work will investigate the structural and functional properties of CnoX proteins expressed by more distant bacteria, it will also be interesting to address the questions that remain open regarding *Ec*CnoX and *Cc*CnoX. For instance, it remains unclear whether chlorination induces conformational changes in the TPR domain of *Ec*CnoX and how de-chlorination occurs *in vivo* after stress. Future research will also determine whether the function of *Ec*CnoX is limited to the defense mechanisms against HOCl or if this protein is involved in other cellular processes. The ability of CnoX to cooperate with the GroEL/ES nanomachine, which was apparently conserved during evolution, is the second major property of CnoX proteins that deserves to be further explored. Here, it will be important to identify the structural features of CnoX chaperedoxins that allow them to transfer substrate proteins to GroEL/ES and to determine whether these features are found in other bacterial holdases. Whether the reported interaction between CnoX and GroEL (Lin and Wilson, 2011) is functionally relevant will also be determined. Finding out how CnoX recognizes its substrates and what is the role, if any, played by the TPR domain in controlling substrate selectivity are other outstanding questions.

## Author Contributions

The authors contributed equally to the redaction. Both authors contributed to the article and approved the submitted version.

## Conflict of Interest

The authors declare that the research was conducted in the absence of any commercial or financial relationships that could be construed as a potential conflict of interest.
